# Female mice respond to male ultrasonic ‘songs’ with approach behaviour

**DOI:** 10.1098/rsbl.2009.0317

**Published:** 2009-06-10

**Authors:** K. Hammerschmidt, K. Radyushkin, H. Ehrenreich, J. Fischer

**Affiliations:** 1 Cognitive Ethology, German Primate Center, Göttingen, Germany; 2 Division of Clinical Neuroscience, Max-Planck-Institute of Experimental Medicine, Göttingen, Germany

**Keywords:** ultrasonic vocalizations, playback experiments, mice, courtship behaviour, female preference

## Abstract

The ultrasonic vocalizations of mice are attracting increasing attention, because they have been recognized as an informative readout in genetically modified strains. In addition, the observation that male mice produce elaborate sequences of ultrasonic vocalizations (‘song’) when exposed to female mice or their scents has sparked a debate as to whether these sounds are—in terms of their structure and function—analogous to bird song. We conducted playback experiments with cycling female mice to explore the function of male mouse songs. Using a place preference design, we show that these vocalizations elicited approach behaviour in females. In contrast, the playback of pup isolation calls or whistle-like artificial control sounds did not evoke approach responses. Surprisingly, the females also did not respond to pup isolation calls. In addition, female responses did not vary in relation to reproductive cycle, i.e. whether they were in oestrus or not. Furthermore, our data revealed a rapid habituation of subjects to the experimental situation, which stands in stark contrast to other species' responses to courtship vocalizations. Nevertheless, our results clearly demonstrate that male mouse songs elicit females' interest.

## 1. Introduction

During female–male courtship encounters, male mice produce ultrasonic vocalizations (Holy & Guo 2005; Jamain *et al*. 2008). As mice are an important model species for studying the genetic basis of behaviour via *knock-in* and *knock-out* technology, also with respect to human disorders, their vocalizations have been recognized as important readout of communication skills (Groszer *et al*. 2008; Jamain *et al*. 2008).

Based on acoustic analysis of temporal and spectral features, Holy & Guo (2005) examined the structure of male mouse ‘songs’. They were able to sort the structurally highly variable call elements into a few discrete categories. Moreover, they showed that the temporal sequence differed significantly from a random pattern and, within sequences, found preferred transition probabilities to different types of call elements. Based on these findings, they suggested that male mouse courtship vocalizations are structurally similar to bird songs and proposed that they serve similar purposes (Holy & Guo 2005).

Here, we explore whether male mouse songs have a function analogous to bird song. In birds, song serves to mark and defend territories and to attract females (e.g. Catchpole & Slater 2008). To assess the influence of male mouse song on female mice, we conducted playback experiments to test whether the sounds alone are sufficient to elicit approach behaviour. As control sounds, we presented ultrasonic pup vocalizations and artificial sounds. We determined the reproductive cycle stage of each female and tested all once during oestrus and once in dioestrus. We predicted that control stimuli should not evoke approach behaviour, since a previous study demonstrated that only lactating females respond to playback of pup sounds (Uematsu *et al*. 2007).

## 2. Material and methods

### (a) Animals

Experiments were performed with 32 female C57BL/6NCrl (Charles River Laboratories, Sulzfeld, Germany) mice. Animals were housed at four to five per cage in a room with 12 L : 12 D cycle (lights on at 09.00) and ad libitum access to food and water. Age of mice at beginning of testing was 12–15 weeks. Females had no contact with males before the experiments. Behavioural tests were conducted during the light phase from 10.00 until 17.00. All experiments were performed with permission of Bezirksregierung Braunschweig (local Animal Care and Use Committee) in accordance with German Animal Protection Law.

### (b) Oestrus determination

For the two weeks prior to experimentation, 40 female mice were monitored daily for oestrus cycle stage by means of vaginal smears. Eight females were excluded owing to irregular cycles. Smears were taken by saline lavage (Bingel & Schwartz 1969) at 10.00. Cell types were identified in unstained wet preparations, and oestrus stages categorized as follows (Allen 1922): (i) dioestrus: many leucocytes and a few nucleated epithelial cells; (ii) proestrus: largely small, round, nucleated epithelial cells, singly or in sheets. None-to-few leucoytes; (iii) oestrus: hundreds of large cornified cells (squames) with degenerate nuclei and masses of adherent cornified cells; and (iv) metoestrus: many leucocytes and a few cornified cells.

### (c) Experimental apparatus

**Figure 1.**
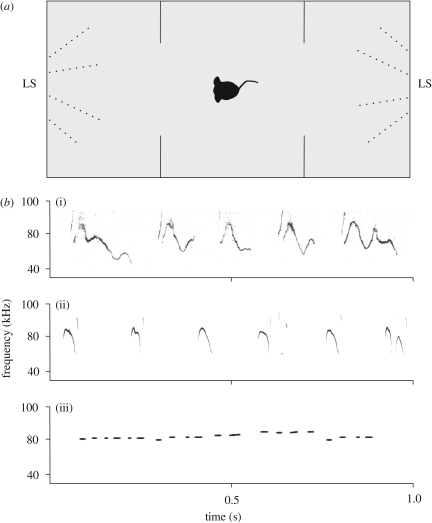
(*a*) Sketch of the experimental setup. LS, loudspeaker. (*b*) Examples of playback sounds: (i) male vocalization recorded during female male encounters (C57BL/6NCrl), (ii) pup vocalization recorded during short-term isolation of 4-day-old pups (other strain C75BL/6) and (iii) artificial short ultrasounds ranging between 70 and 80 kHz (generated with Avisoft SASLab Pro v. 4.33). Recording equipment: Microphone (UltraSoundGate CM16), interface (UltraSoundGate 116), recording software Avisoft SASLab Pro v. 4.33, sampling frequency 300 kHz; artificial sounds were produced with Avisoft SASLab Pro v. 4.33.

Mice were tested for place preference in a rectangular, three-chambered box. Each chamber was 20 cm × 40 cm × 22 cm in size. Dividing walls were made of clear Plexiglas, with openings (10 cm) in the middle allowing access into each chamber and proper propagation of ultrasound. Loudspeakers were situated in front of the outer walls of the peripheral chambers, which had corresponding circular openings with stainless-steel mesh insets for free propagation of ultrasound. Two identical loudspeakers (one active and one silent) were visible during place preference measurements (figure 1*a*). The side of playback was pseudo-randomized and counterbalanced. The test apparatus was cleaned with water after each subject.

### (d) Behavioural testing

We tested 32 female mice in random order during the oestrus and dioestrus phase (i.e. each mouse was ultimately tested twice with 2–3 days between trials). Three different types of playback sounds (figure 1*b*) were broadcast for three separate groups of females: (i) male mouse song (*n* = 10), (ii) pup isolation calls (*n* = 11), and (iii) whistle-like control sounds (*n* = 11). The maximum root mean square (RMS) of sound pressure level (SPL) was 81.1–84.0 dB measured at 0.2 m (sound level metre: Brühl & Kjær 2231). The SPL of playbacks was comparable with the SPL of natural male vocalizations, which we could confirm by similar microphone distance and recording gain. We applied a bandpass filter to cut off frequencies below 40 kHz. All playbacks were broadcast at a sample rate of 192 kHz. For playbacks, we used a notebook and ultrasound playback interface with integrated D/A converter and power amplifier (UltraSoundGate Player 116). The interface was connected to an Ultrasonic Dynamic Speaker ScanSpeak. To control the correct playback transmission and to record possible female sounds, we recorded each session using Avisoft SASLab Pro v. 4.33 at a sampling frequency of 300 kHz. Microphone (UltraSoundGate CM16) and interface (UltraSoundGate 116) with pre-amplifier and A/D converter were connected to a notebook (ultrasound hardware and software: Avisoft Bioacoustics, Berlin, Germany). The microphone was placed above the middle compartment of the place preference box.

At session start, we placed the female mouse in the middle compartment of the place preference box. Three minutes later, we broadcast the playback sound lasting 60 s. After the end of presentation, we continuously observed the subject's behaviour in the box for another 3 min. The behaviour was recorded by an overhead video camera and video-tracking software (‘Viewer 2’, Biobserve GmbH, Bonn, Germany) to calculate the time spent in each chamber. SPSS 15.0 was used for the statistical analysis.

## 3. Results

**Figure 2.**
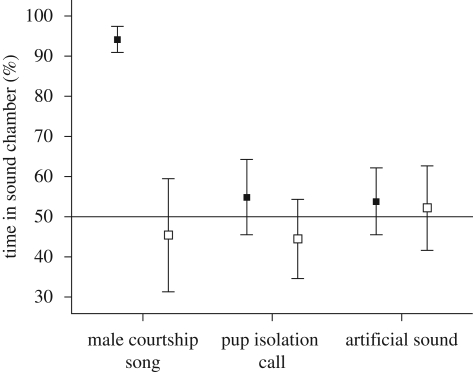
Percentage of time in sound chamber (mean ± s.e.m.). Line shows chance level. Filled squares, first presentation; open squares, second presentation.

A mixed linear model with ‘type of playback sound’, ‘oestrus state’ and ‘presentation order’ as fixed factors and subject as a random factor revealed a significant difference for presentation order (*F*_1,46_ = 7.2, *p* = 0.01) and a significant interaction between the type of playback sound and presentation order (*F*_2,46_ = 3.9, *p* = 0.027). The other factors revealed no significant differences (type of playback sound: *F*_2,46_ = 1.59, *p* = 0.22; oestrus state: *F*_1,46_ = 0.89, *p* = 0.42). Owing to the significant interaction, it was necessary to conduct separate tests for the first and second playback presentation. We found that, in the first presentation, females spent significantly more time at the side from which male mouse songs were broadcast than in the control conditions (*F*_2,27_ = 8.8, *p* = 0.001; figure 2). No such preference was found in the second presentation (*F*_2,21_ = 0.23, *p* = 0.8). Responses to other playback sounds remained independent of presentation order. In addition, we found no significant differences whether females were in the oestrus phase or not (first presentation: *F*_1,27_ = 0.18, *p* = 0.9, second presentation: *F*_1,21_ = 0.63, *p* = 0.54).

## 4. Discussion

The playback study showed that female mice are attracted to sounds of male songs, whereas they neither displayed a preference nor avoidance of pup vocalization or artificial sounds. Surprisingly, there was a rapid habituation to the male songs, and the positive response was independent of the female reproductive cycle stage. These results are in line with an earlier study by Pomerantz *et al*. (1983), which showed that females prefer intact vocalizing males over devocalized males. Females also preferred devocalized males that were presented along with playbacks of synthetic ultrasonic vocalizations over devocalized males paired with silence. In these experiments, females preference was unaffected by the oestrus stage.

The attraction of female mice to male songs corresponds to findings from playback studies in birds and other species. Field playback experiments and laboratory studies in birds demonstrated that male song causes females to approach the sound source (Baker *et al*. 1981; Eriksson & Walin 1986). Likewise, in tree frogs (Gerhardt & Schul 1999) or crickets (Doherty & Storz 1992), playbacks of male songs are sufficient to elicit approach behaviour towards the loudspeaker. In tree frogs (Gerhardt & Schul 1999) as well as in female birds (Catchpole *et al*. 1984), the positive phonotaxis depends on the reproductive state of females. In female white-throated sparrows, the selective response to species-specific songs is dependent on oestradiol levels (Maney *et al*. 2008). In this species, songs that attract females during the breeding season function as aggressive signals for defending food resources during the non-breeding season. Similar interactions between social signals and hormone level exist in rodents. In rats, new mothers lick and nurse pups, whereas virgin females find pups aversive. The behaviour towards pups can be altered by hormonal manipulation (Rosenblatt 1975). In this respect, it is surprising that in our study the female response to male courtship vocalizations was independent of oestrus.

That female responses were independent of cycle stage raises questions about the function of these signals. Instead of being used to attract mating partners, it seems more likely that these songs facilitate mating, maybe by stimulating females or causing females to stay in close contact. This view is supported by behavioural studies comparing ultrasonic vocalizations and related behaviour of females and males. Sales (1972) found that male mice began to utter ultrasonic vocalizations immediately or shortly after subjects were placed together with females and observed a peak of ultrasonic signal emission during investigative behaviour of males. During mating, the rate of ultrasonic calls was reduced. Between mating events, when mice are no longer directly engaged, ultrasonic calls were only sporadically detected (for detailed discussion, see Nyby 2001). Further studies on free-ranging animals would be desirable to assess the function of these signals under more natural conditions.

Females showed a rapid habituation to male songs. A 1 min playback trial was sufficient to reduce the response in the second trial 2–3 days later. In birds and other species, playbacks can be used repeatedly to attract females without strong habituation (Byers & Kroodsma 2009). The observed habituation here is unlikely to be related to repeated presentation of ultrasonic sounds. Playback tests with lactating female mice have shown that ultrasound playbacks can be presented repeatedly without immediate habituation (Ehret & Haak 1982). Similarly, juvenile and adult rats responded repeatedly with approaches to 50 kHz sounds of adult males (Wöhr & Schwarting 2007). The rapid habituation found here, although remaining an issue for further investigation, has important implications for future experiments in that repeated measure designs should be avoided.

Collectively, our results indicate that playback studies employing a place preference paradigm can be used to assess differential responses to different ultrasonic stimuli. Since the effect of specific genes not only on vocalization but also on perception and processing of vocal signals is of major interest, this method may help assessing behaviour of genetically modified mice.
